# The Architecture of Decolonial Partnerships in University Global Health Program Development

**DOI:** 10.5334/aogh.4952

**Published:** 2026-02-17

**Authors:** Stephanie Crane, Alfredo Hernandez Moralez, Wendys Filpo Diaz, Babs Waldman, David Ansell, Ernhis Montero Hernandez, Jessica Vlaming, Kelly Dressel, Sophie Young, Zoe Kusinitz

**Affiliations:** 1 Rush University Medical Center, Chicago, IL, USA; 2 Community Empowerment, Buenos Aires, Dominican Republic

**Keywords:** decolonialization, academic medical center, non-governmental organization, program development, university partnerships, clinical services, global scholarship

## Abstract

*Background:* Processes and best practices for initiating and growing university global health programs in high-income countries (HICs) synchronously and symbiotically with partners in low- and middle-income countries (LMICs) are not abundantly described in the medical literature. In particular, programs that do not have university partners in LMICs may struggle to develop sustainable, ethical, and anticolonial community and governmental partnerships.

*Methods:* This article reviews existing literature and describes the challenges in the contemporaneous development of university global health programs and community/governmental partners. The paper goes on to describe the creation of the Office of Global Health at Rush University in conjunction with the inception and development of its partner non-governmental organization (NGO), Community Empowerment in the Dominican Republic. The success and opportunities in the evolution of this ongoing relationship are described. Guiding principles for others attempting similar work are provided.

*Results:* Creating these entities simultaneously promotes the establishment of relationships with equal power and authority from the inception, facilitates the creation of customized programs that capitalize on the strengths of the university and infrastructure of the partner country/community, and allows both entities to grow together in scope and impact. Challenges include identifying and nurturing like-minded university, NGO, and community/government partners; securing bilateral sustainable funding; ensuring quality of clinical services and educational/scholarly activities; and consistently promoting anticolonial practices.

*Conclusion:* Developing university global health programs in HICs simultaneously with a partner NGO can result in mutual and commensurate growth and outcomes as well as strong and equitable relationships. This paper describes the author’s own experience at Rush University building connections with community partners and colleagues in the Dominican Republic and outlines strategies to achieve these results.

## 1. Introduction

The modern understanding of the importance of global collaboration in medical practice, education, and research has been well established. However, to understand the history of the field of “Global Health,” is to understand the history of colonialism. From early colonialism in the 15th–17th centuries, characterized by early European exploration and the accompanying deadly diseases, to the Social Darwinism of the 19th century, to the medical missionary era, and into the current neo-colonial landscape characterized by economic imperialism and uneven wealth distribution, the field of “Global Health” remains rife with colonial vestiges [1, 2]. While modern definitions of global health focus on eliminating healthcare disparities wherever they exist, be that in the Global South or the Global North, these definitions are also interwoven with and defined by powerful stakeholders and economic interests [3].

The process of establishing and maintaining decolonial academic global health programs remains a challenge that is both existential and pragmatic, driven by complicated factors, such as the geopolitical climate, resource limitations, and socio-cultural biases [4, 5]. The power imbalance driven by the reality that the Global North most often wields fiscal authority is inherently a colonial practice [6]. From the inception of a collaboration, both parties must work actively and diligently to mitigate this wealth and power inequity [7]. This problem is accentuated in small academic centers in high-income countries (HICs) that may lack existing university partnerships in low- and middle-income countries (LMICs). The financial base supporting program development initiatives must be systematically developed through grants and philanthropy, most of which flow unilaterally from HICs to LMICs [7, 8]. Thus, it is of particular importance that relationships between HIC’s global health programs and partners in LMICs be constructed from the onset with equity, respect, and mutual benefit [9]. A true measure of a successful program would be to work itself out of a job. Establishing these longitudinal relationships prevents the short-term experience in global health (often referred to as STEGH) scenario, which results in minimal long-term impact and can raise false expectations when not embedded in a sustainability framework [10].

The intersectionality between academic global health offices and global non-governmental organizations (NGOs) has not been well studied, and little has been published in the literature regarding the value of growing these entities contemporarily and symbiotically. The body of previous literature reveals that many global health programs affiliated with health science colleges and training programs rely on existing NGOs and/or global university partners to provide platforms for service learning and scholarship [9]. While this approach has some advantages, small programs in their inception might not have these opportunities. Furthermore, the benefits that arise from growing both entities synchronously are substantial, as the organic nature of this growth allows for capitalization of individual strengths and capacities.

The objective of this article is to describe the creation of an Office of Global Health at Rush University in conjunction with Community Empowerment, an NGO licensed in the Dominican Republic and the United States, and to illustrate with specific site descriptions the process of creating non-dependent programs and sustainable care. This paper will describe important experiences building these relationships at Rush, as well as discuss program implementation steps and lessons learned.

## 2. Methodology

**Figure 1:**
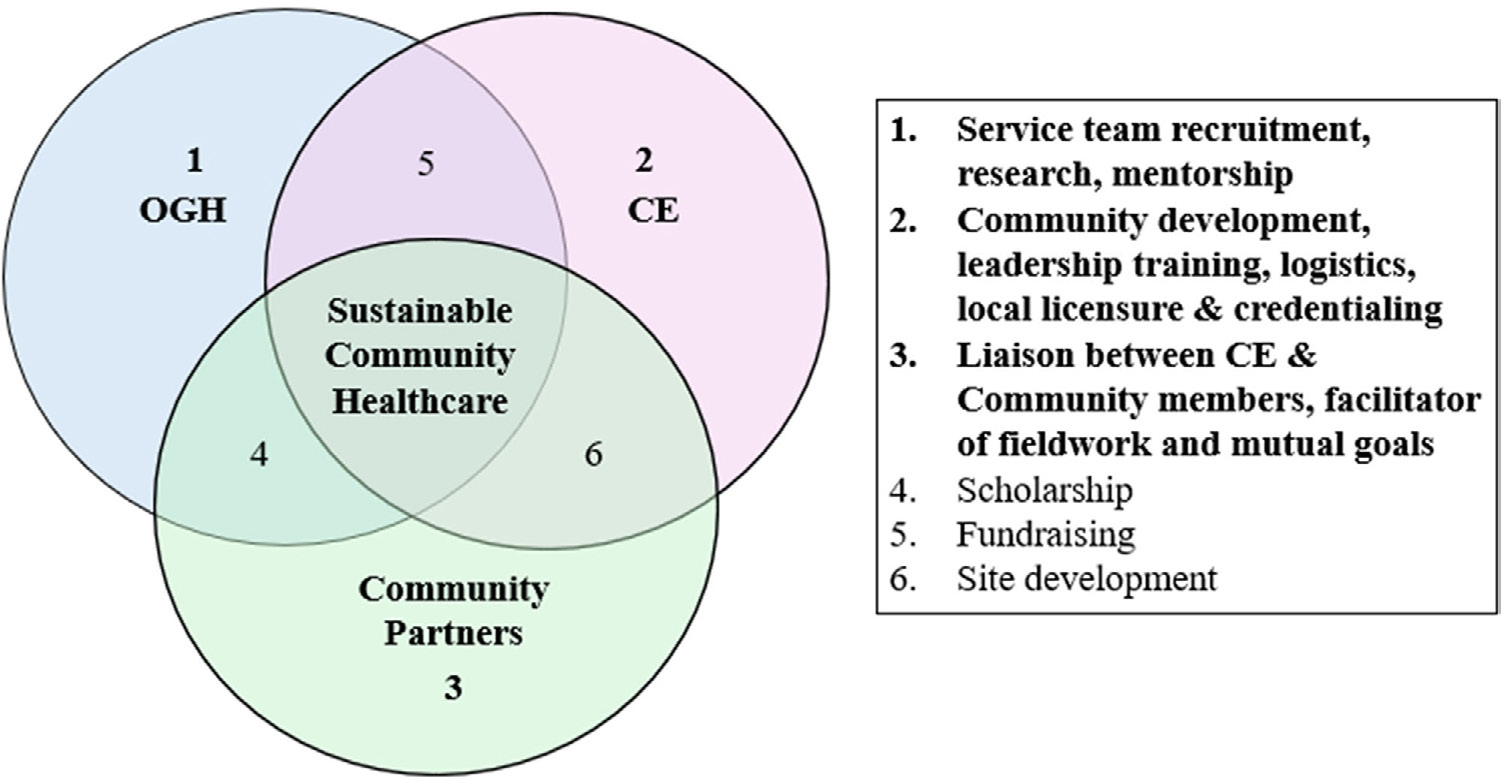
Structural overview of individual and shared duties of members of the sustainable community healthcare partnership.

Taking an anticolonial approach with a focus on building genuine, equitable relationships resulted in the development of a platform that fosters sustainable and reproducible healthcare services. The Office of Global Health at Rush works with Community Empowerment to bring primary care and public health initiatives to medically under-resourced communities in the Dominican Republic. While distinct legal entities, these organizations work symbiotically toward the common goals of improving health and exposing medical trainees to ethical methods and outcomes. Figure 1 highlights the general activities under the purview of each entity and their intersectionality, resulting in sustainable healthcare in the partner community.

### Establishment of an academic office of global health

The Office of Global Health at Rush was founded in 2012, although many Rush faculty members and trainees volunteered their medical and surgical services in the Dominican Republic as early as 2005. Founding members included an MD/Director and a Program Manager, with a combined 1.6 full time equivalent (FTE). The Office of Global Health has since grown to additionally employ administrative and research assistants for a combined 2.65 FTE. The mission of the Office of Global Health is to provide opportunities for trainees and faculty to participate in clinical and scholarly activities that contribute to the sustainable reduction in health disparities. The office recruits provider-volunteers, students, and residents from all three colleges at Rush University and multiple residency training programs. These trainees are integrated into teams, where they assist with supervised care provision, participate in scholarly projects, or perform research in collaboration with Dominican community members and students. In addition, the department assists with grant writing, supply sourcing, and philanthropic outreach. This provides for financial needs such as trainee scholarships, medical and surgical supplies, and infrastructure development funding.

### Establishment of NGO

Community Empowerment was officially founded in 2016, although Dominican community volunteers had been working with volunteer medical and surgical providers from RU since 2005. Community Empowerment works with Dominican communities and community leaders who have identified gaps in both personal and public health services and are willing and able to be equitable partners in the process to improve health. This process of community engagement, developing trust, and leadership identification and development takes several years. A community needs assessment is conducted early in the process to better understand the most pressing personal and public health issues.

During this community development process, direct primary adult and pediatric care is provided on a quarterly basis by medical teams from Rush and affiliated programs. Chronic disease management is delivered, and medications are dispensed quarterly with three months of medication provided. In addition, acute care services are delivered, and an electronic medical record is used to document and surveille patient care, assess quality, and measure outcomes. Healthcare education is provided by both Rush teams and local experts. Public health gaps are addressed through the creation of potable water plants and additional infrastructure improvements. An example includes addressing mosquito-borne illnesses in our partner community of Villa Verde via an assessment of standing water and partnering with Engineers Without Borders (EWB) to assist with the reduction of flooding and water stagnation. Synchronous to the community development process and direct care provision, funds are raised via grants and philanthropy to purchase land and build a clinic facility.

### Transition to sustainability

**Figure 2:**
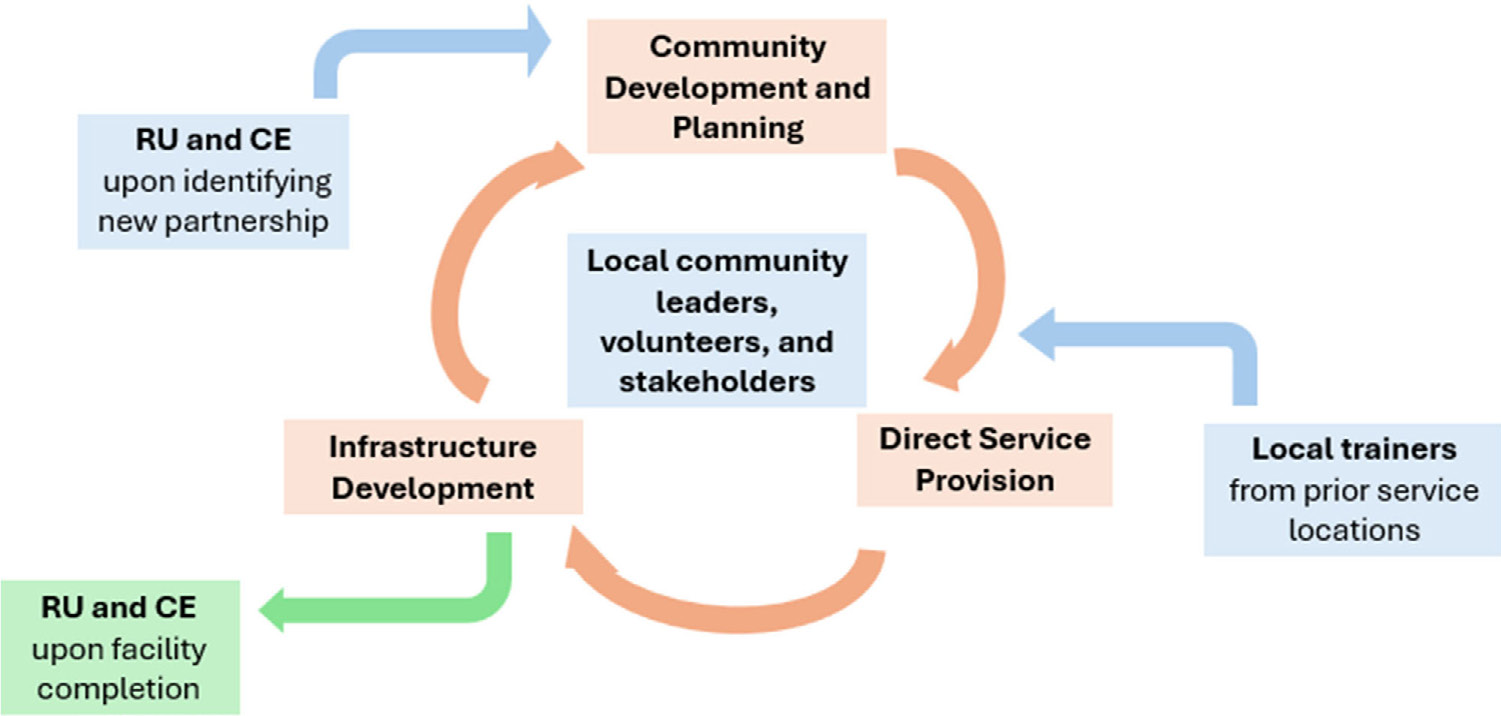
The cycle from community development to sustainability.

Upon completion of the clinic facility, it is given to the Ministerio de Salud Publica y Asistencia Social (MSP) of the Dominican Republic under a legally binding contract that requires the MSP to staff and supply the clinic in perpetuity in exchange for ownership of the land and clinic facility. Clinical staff, including at least one physician and nurse, several community health workers, and a pharmacist, are recommended by the community to the MSP for employment in the clinic. Individuals who have previously volunteered with CE are prioritized. The water purification plant provides clean water as well as local jobs and is managed locally. At this point, the cycle depicted in Figure 2 is considered complete, and medical teams from Rush no longer provide direct clinical care. The complete integration of clinical services into the MSP assures longevity and bolsters local medical infrastructure instead of competing with it.

### Key components for replicability

In order to scale this model, certain elements of each entity—the supporting academic program, the local NGO, the community leaders and volunteers, and the local MSP—are necessary to ensure longevity and success. The academic partner must effectively recruit volunteer healthcare providers and develop programming for residents and students to allow for protected time and credit. Additionally, the academic partner must acquire philanthropic support to cover surgical supplies, medications, trainee scholarships, and infrastructure development projects until clinics have been fully integrated locally. The local NGO must be capable of navigating local politics and obtaining medical provider licenses/privileges, as well as leading community development efforts and managing volunteer medical team logistics. Communities must identify leaders who are motivated and mission-aligned with their academic partner, as well as engage volunteers who are willing and able to support the local activities. A local Ministry of Health equipped with adequate infrastructure and resources to assume management and financial support of clinics once they have been integrated is essential.

### Establishment of additional services

In addition to gaps in primary and advanced medical care, significant gaps in surgical care often exist in LMICs. These gaps are pronounced in public health facilities due to insufficient financial resource allocation for physicians working within the public healthcare system. Thus, these needs in Rush University’s partner communities are addressed by one-week multi-specialty surgical teams from Rush University Medical Center and affiliates. Primary care patients with elective surgical needs are referred to surgical teams on a continuous basis, and data are collected assessing quality and outcomes. Surgeries are performed in three partner hospitals in the Azua Province.

Care is taken to bring all supplies necessary to operate without depleting local resources. Postoperative care is provided by local providers, and local medical students and residents are integrated into surgical teams alongside Rush trainees. Follow-up care is provided by local surgical partners. Partner hospital infrastructure is enhanced by collaborations with organizations, such as Project C.U.R.E., which provides shipping containers of repurposed equipment and supplies. This approach fosters local infrastructure development instead of resource competition.

## 3. Results

### Primary care services

Due to the successful transition of the Peralta Clinic to an independently functioning program, data are no longer available through the Office of Global Health/Community Empowerment. The MSP is now responsible for collecting data. Table 1 outlines the site-specific metrics of the two subsequent communities, Villa Verde and Duquesa.

**Table 1:** Site-specific patient metrics.

TOTAL SITE-SPECIFIC NUMBER OF PATIENTS SEEN YEARLY (FY23–25)			
**Villa Verde site**	2022–2023	2023–2024	2024–2025
**Patient metrics**			
**Total patient visits**	1280	1339	1656
**Total number of patients**	779	882	897
**Duquesa site**	2022–2023	2023–2024	2024–2025
**Patient metrics**			
**Total patient visits**	1772	1513	1255
**Total number of patients**	830	1000	901

### Surgical services

**Figure 3:**
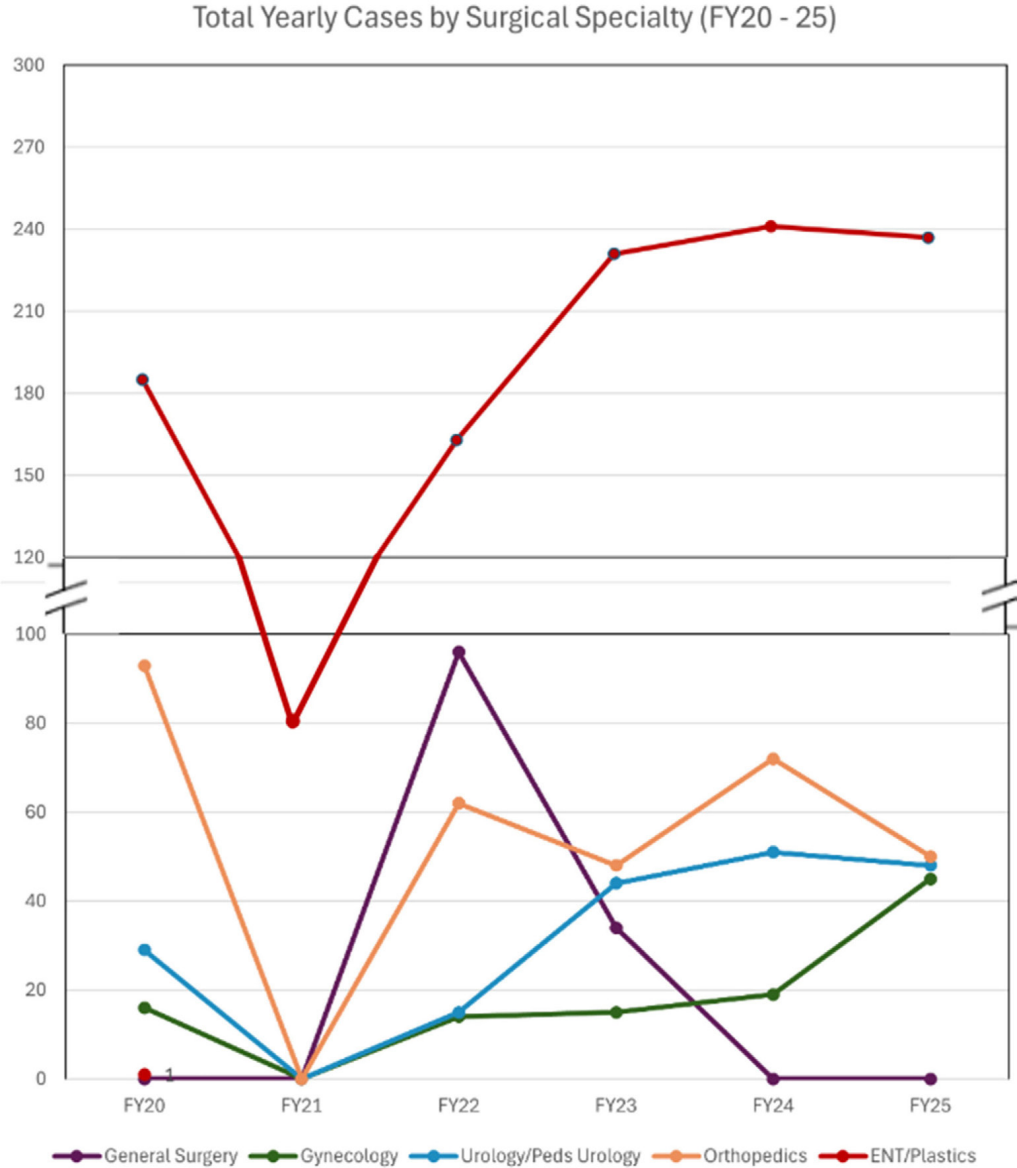
Total yearly cases by surgical specialty (FY2020–current). To note, FY21 case numbers are limited by trip cancellations due to the COVID-19 pandemic.

Figure 3 illustrates total yearly surgical cases by specialty, from 2020 to 2025. All cases during the COVID pandemic decreased and then rebounded. General surgery has remained low due to a decline in surgical volunteers. However, a general surgery trip is scheduled for the spring of 2026. Otolaryngology numbers remain markedly higher, with three trips per year due to the very high level of need. There are no public Dominican otolaryngologists operating in the Southwest part of the country.

**Figure 4:**
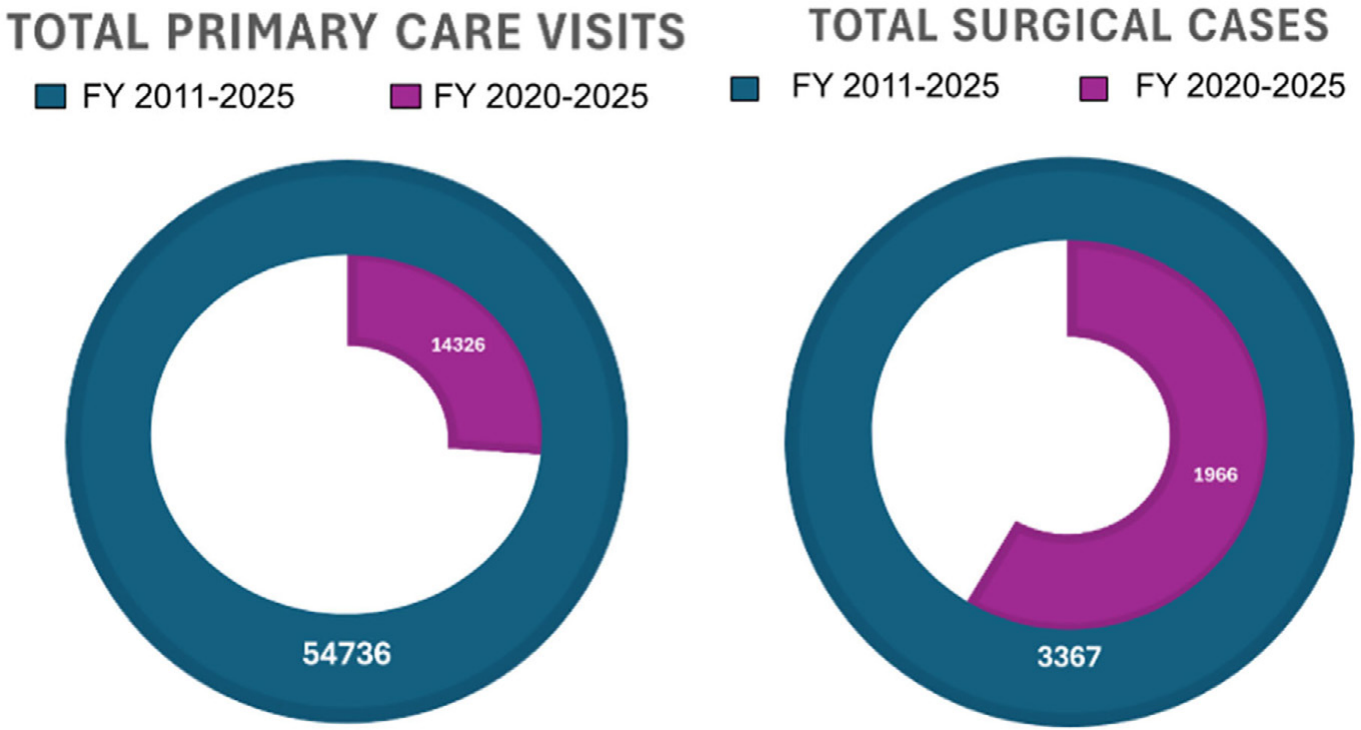
Overview of clinic volume over a fourteen-year period versus the most recent five-year period.

Figure 4 demonstrates the total number of primary care visits and surgical procedures from 2020 to2025, as compared against the total number of cases from 2011 to 2025. The number and size of primary care teams have remained stable, while the expansion of surgical programs resulted in faster growth.

### Volunteers

Table 2 provides percentages of volunteers per role from 2020 to 2025. Of note, as the programs have grown and resident tracks have been developed in various specialties, the relative proportion of healthcare providers to trainees has decreased, with the ratios being relatively stable over the last three years.

**Table 2:** Table of volunteer participation count by role from 2020 through 2025.

TOTAL VOLUNTEER COUNT BY ROLE (2020–CURRENT)					
**Duquesa & Villa Verde sites**	2020–2021	2021–2022	2022–2023	2023–2024	2024–2025
**Healthcare providers (doctors, dentists, pharmacists, audiologists)**	67.65%	40.65%	30.25%	30.53%	27.57%
**RNs**	17.65%	13.82%	10.49%	12.63%	12.15%
**Residents**	2.56%	8.13%	20.37%	18.95%	16.82%
**Students**	2.56%	7.32%	22.22%	16.84%	14.95%
**Non-medical (high school students, logistical staff, artists)**	5.88%	8.13%	15.43%	10%	8.41%
**Support staff (Scrub tech, MAs, PCTs)**	2.56%	6.50%	2.47%	2.11%	0.94%
**Total volunteers**	34	123	162	190	214

*In 2020–2021, the number of volunteers was significantly lower due to the COVID-19 pandemic affecting the participation of volunteers.

### Scholarship

Table 3 provides examples of scholarly projects completed by students in various health science disciplines and residencies. Whenever possible, interdisciplinary groups are formed, as illustrated by the Buckman et al. study. In addition, every attempt is made to include Dominican medical students and residents in studies. The Dressel project incorporated several local participants and authors.

**Table 3:** Examples of scholarly global health projects.

SCHOLARLY GLOBAL HEALTH PROJECTS		
Student & resident names	College in Rush University/role	Citation
Victoria Buckman, Samiya Diawara, and Morgan Sturgis et al.	Medical College students and the IM Residency program	Sturgis et al. [11]
Kelly Dressel	College of Nursing	Dressel [12]
Megan Beiler	College of Health Sciences	Beiler [13]
Nicholas Tasiopoulos	College of Health Sciences	Tasiopoulos [14]

### Independent communities

#### Peralta

The first site in the rural community of Peralta took approximately ten years to complete the cycle towards independence. A local physician, nurse, and pharmacist were hired, and several long-time volunteers were trained as community health workers. The lengthy cycle was due to difficulty in identifying a sustainability strategy. Many community members live on a dollar or less per day and have no ability to contribute financially to healthcare. Several local businesses were started with the goal of floating revenue back to sustain clinic operations, but the profit margin was too meager. Ultimately, the combination of the revenue from the water plant and the contract with the MSP allowed the clinic to become fully financially independent. Six years after this cycle was completed, both the clinic and water plant are open and providing services.

#### Villa Verde

Five years from inception, the second site in the community of Villa Verde has signed a contract with the MSP, and the clinic has been fully transitioned. A local physician, two “pasantes” (recent medical graduates completing a year of service), one nurse, one pharmacist, and several community health workers have been hired and are providing care. The Villa Verde water purification center, which provides clean water locally, has been launched and is managed by a community organization. The area continues to be supported by community health initiatives, such as the EWB partnership described above.

#### Duquesa

Work has begun in the third community of Duquesa and is in its third year. This community sits on the largest landfill in the Caribbean and is largely inhabited by undocumented Haitian immigrants who collect and sell garbage. Dominican law prevents undocumented Haitians and their descendants from achieving Dominican citizenship; thus, this community is effectively excluded from most public services, including healthcare [15]. Here, the model of the clinic to the MSP to ensure longevity and sustainability is not possible. Given the ongoing need for healthcare services, Community Empowerment is in the process of transitioning care of this community to another NGO indefinitely. A needs assessment conducted by Rush medical students revealed a strong need for contraception and women’s health education, leading to a partnership with the NGO ProFamilia for the delivery of Depo-Provera and Pap smears. A program to implement a train-the-trainer model to empower women from the community to deliver sexual health education was successfully designed and implemented by a Rush trainee. This program remains active, with female leaders from the community maintaining the educational sessions.

#### Oregano Grande

**Figure 5:**
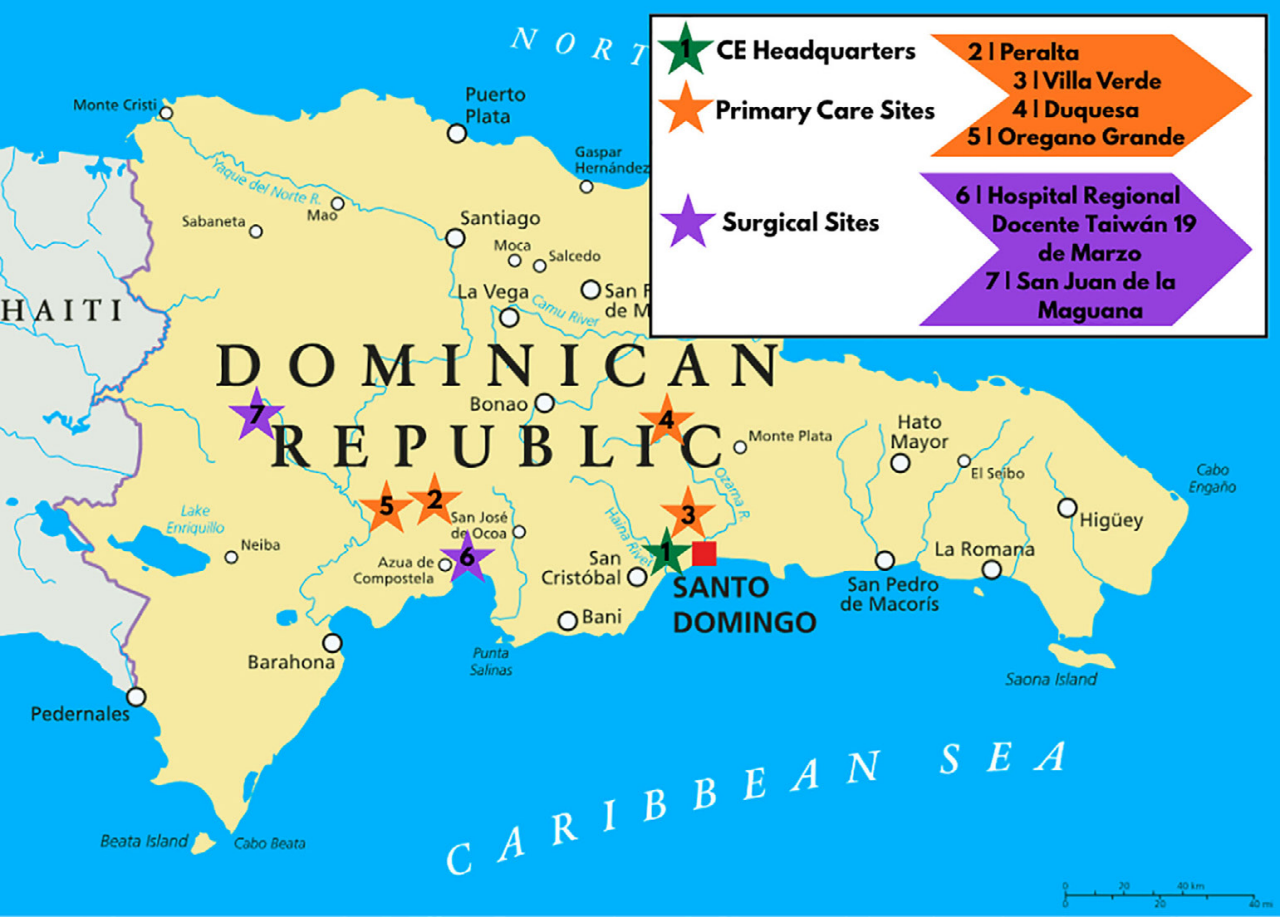
Map of surgical, primary care, and headquarters sites across the Dominican Republic.

The pilot team to the community of Oregano Grande is planned for August 2025, and a community assessment is scheduled for October 2025. Figure 5 outlines the distribution of surgical, primary care, and philanthropic sites across the Dominican Republic, including this new site.

## 4. Discussion

### Key findings

Our findings reveal that much of the literature in this sector comes out of well-funded programs or large universities (≥15,000 students) and their global university partners. Scant literature exists on the values, implementation, risks, and benefits of university-based global health programs growing their global platforms and strategies alongside global health-focused NGOs in an ethical and mutually beneficial manner.

Rush University, while small by the number of matriculated students (<5000), is nationally recognized for its strong multidisciplinary health education. Rush University Medical Center is esteemed for its excellence in clinical care and strong equity mission. Growing out of these strengths, the Office of Global Health at Rush and its partner NGO, Community Empowerment, were able to mutually scale their impact.

Although legally distinct, we found that growing these entities together via robust bidirectional relationships has positively impacted outcomes in clinical, educational, and scholarly arenas while ensuring a decolonial approach with sustainability endpoints. Mutual scaling has maximized efficiency and conserved resources, which is particularly relevant in a resource-scarce environment. Creating these entities simultaneously promotes the establishment of relationships with equal power and authority from the inception, facilitates the creation of customized programs that capitalize on the strengths of the university and infrastructure of the partner country/community, and allows both entities to grow together in scope and impact. It is important to note that growth occurred slowly, beginning with one community, and that organic growth prevented financial overreach.

### Challenges

#### University

A significant challenge faced in the establishment of novel global health programs is often the lack of a definitive and reliable funding source [16]. Thus, presenting the indirect financial benefits of such a program to university leadership is paramount. There is ample evidence from the literature that trainees in healthcare fields desire and factor in the existence of global health experiences in their selection of training programs [17].

Our anecdotal experience at Rush has confirmed this pattern. Trainees routinely express that their selection of Rush was contingent on the presence of an Office of Global Health, even if the trainee never actually participates in global Health programs. In addition to trainees, offering faculty an opportunity to participate in ethical global health experiences can enhance well-being and motivation, positively affecting their work at home and combatting burnout [18]. We have found that Rush faculty value the opportunity to volunteer in vetted sites, work with their own residents and students, and customize their schedules and time commitments.

Relying on philanthropy for connections with potential donors/grants can be challenging, as there is often competition for these dollars for needs at home. At Rush, we were able to connect with a few donors interested in global health disparities through the Office of Philanthropy, resulting in several impactful grants. To increase competitiveness for funding, highlighting the educational benefit of exposure to diseases not endemic to the United States to university leadership in HICs can be impactful in the current era of expanding globalization.

#### Partner NGO

There are many challenges faced by the partner NGO. Perhaps the most important decision to be made is the selection of a partner community. Identifying a community that has strong leadership, has clear and attainable goals for improving the health of its population, and understands that healthy relationships share power and responsibilities is not a process that happens overnight. In addition, the process of transitioning a clinic into the MSP system has some risk, as a contractual agreement has proven to be only as good as the health minister in power at the time and needs to be regularly renegotiated.

Another significant challenge is the temptation to assume that what worked in one community will automatically work in another. We have learned that while elements such as those mentioned above are indeed foundational, they may play out quite differently in each community. As described above, the process of integrating a clinic into the MSP system was not possible in Duquesa, and another solution was identified.

### Future implications

A successful model for implementing global health programs has been described, with the potential for replication in universities with similar resources and partnerships. This case study provides novel insights and recommended practices for creating equitable, sustainable, and decolonial global health programs at academic medical institutions. Key elements to success include capitalizing on existing institutional strengths, demonstrating value-added to institutional leadership in order to secure baseline funding, and identifying global partners who have both vision and capacity to improve health in their communities.

## Additional File

The additional file for this article can be found as follows:

Supplementary Materials 1. Community empowerment website: https://community-empowerment.org/.
