# Supplementary material for: The Architecture of Decolonial Partnerships in University Global Health Program Development
Source: Ann Glob Health. 2026 Feb 17;92(1):17. doi: 10.5334/aogh.4952 (PMC12922671; doi:10.5334/aogh.4952)
Supplement: Supplementary Materials 1. — Community empowerment website: https://community-empowerment.org/. [file agh-92-1-4952-s1.pdf]

SUPPLEMENTARY FILE

The additional file for this article can be found as follows:

Supplementary Materials 1. Community empowerment website: https://community-empowerment.org/.
DOI: https://doi.org/10.5334/aogh.4952.s1
